# Differences in Resting State Functional Connectivity between Young Adult Endurance Athletes and Healthy Controls

**DOI:** 10.3389/fnhum.2016.00610

**Published:** 2016-11-29

**Authors:** David A. Raichlen, Pradyumna K. Bharadwaj, Megan C. Fitzhugh, Kari A. Haws, Gabrielle-Ann Torre, Theodore P. Trouard, Gene E. Alexander

**Affiliations:** ^1^School of Anthropology, University of Arizona, TucsonAZ, USA; ^2^Department of Psychology, University of Arizona, TucsonAZ, USA; ^3^Evelyn F. McKnight Brain Institute, University of Arizona, TucsonAZ, USA; ^4^Department of Biomedical Engineering and Department of Medical Imaging, University of Arizona, TucsonAZ, USA; ^5^Arizona Alzheimer’s Consortium, PhoenixAZ, USA; ^6^Neuroscience Graduate Interdisciplinary Program, University of Arizona, TucsonAZ, USA; ^7^Physiological Sciences Graduate Interdisciplinary Program, University of Arizona, TucsonAZ, USA

**Keywords:** functional connectivity, MRI, VO_2max_, physical activity, aerobic exercise

## Abstract

Expertise and training in fine motor skills has been associated with changes in brain structure, function, and connectivity. Fewer studies have explored the neural effects of athletic activities that do not seem to rely on precise fine motor control (e.g., distance running). Here, we compared resting-state functional connectivity in a sample of adult male collegiate distance runners (*n* = 11; age = 21.3 ± 2.5) and a group of healthy age-matched non-athlete male controls (*n* = 11; age = 20.6 ± 1.1), to test the hypothesis that expertise in sustained aerobic motor behaviors affects resting state functional connectivity in young adults. Although generally considered an automated repetitive task, locomotion, especially at an elite level, likely engages multiple cognitive actions including planning, inhibition, monitoring, attentional switching and multi-tasking, and motor control. Here, we examined connectivity in three resting-state networks that link such executive functions with motor control: the default mode network (DMN), the frontoparietal network (FPN), and the motor network (MN). We found two key patterns of significant between-group differences in connectivity that are consistent with the hypothesized cognitive demands of elite endurance running. First, enhanced connectivity between the FPN and brain regions often associated with aspects of working memory and other executive functions (frontal cortex), suggest endurance running may stress executive cognitive functions in ways that increase connectivity in associated networks. Second, we found significant anti-correlations between the DMN and regions associated with motor control (paracentral area), somatosensory functions (post-central region), and visual association abilities (occipital cortex). DMN deactivation with task-positive regions has been shown to be generally beneficial for cognitive performance, suggesting anti-correlated regions observed here are engaged during running. For all between-group differences, there were significant associations between connectivity, self-reported physical activity, and estimates of maximum aerobic capacity, suggesting a dose-response relationship between engagement in endurance running and connectivity strength. Together these results suggest that differences in experience with endurance running are associated with differences in functional brain connectivity. High intensity aerobic activity that requires sustained, repetitive locomotor and navigational skills may stress cognitive domains in ways that lead to altered brain connectivity, which in turn has implications for understanding the beneficial role of exercise for brain and cognitive function over the lifespan.

## Introduction

Expertise and training in motor skills is often associated with changes in brain structure and function (Dayan and Cohen, 2011). Long-term training in activities that require precise fine motor control (e.g., musical training) can lead to structural changes in brain areas that are linked to motor function, as well as in cortical areas involved in sensory, spatial, and attentional processes (Gaser and Schlaug, 2003; Han et al., 2009; Jäncke et al., 2009; Park et al., 2009). Recent work has also shown that expertise in athletic activities that demand high levels of hand-eye coordination, such as golf (Jäncke et al., 2009), gymnastics (Wang et al., 2016), or racquet sports (Di et al., 2012), can alter brain structure and function. However, fewer studies have explored the neural effects of athletic activities that do not seem to require precise motor control (e.g., distance running). Here, we examine potential neuroplasticity associated with expertise in endurance running, a sport that is thought to involve sustained aerobic activity over time with more repetitive rather than complex fine motor skills (Schlaffke et al., 2014).

There is some limited evidence that accomplished endurance athletes have differences in brain structure compared with non-athletes. Highly skilled endurance athletes have enhanced white matter (WM) tracts and greater gray matter volume in the medial temporal lobes compared with more sedentary controls (Schlaffke et al., 2014; Chang et al., 2015). More generally, recent studies have shown that engaging in aerobic exercise (e.g., moderate intensity walking) may alter and improve brain structure and function (Cotman and Berchtold, 2002; Colcombe et al., 2003, 2006; Kramer et al., 2006; Erickson et al., 2011). While much of this work has been focused on older adults, young adults have regionally specific, exercise-related increases in gray matter and WM tract integrity (Marks et al., 2007; Peters et al., 2009; Killgore et al., 2013). Thus, exercise in general, and expertise in endurance athletics specifically, may have important beneficial effects on brain structure and connectivity, with the potential to enhance cognitive resilience over the lifespan.

While exercise-related effects may be the result of non-specific molecular signaling cascades (see Raichlen and Polk, 2013), it is also possible that the cognitive benefits of exercise occur because locomotion is not simply an automated repetitive motor task, but is instead a highly complex behavior that involves domains related to both motor and cognitive functions (Yogev-Seligmann et al., 2008). For example, walking or running through complex environments can engage several components of executive function including volition, self-awareness, planning, inhibition, monitoring, attentional switching and multi-tasking, in addition to motor control (Yogev-Seligmann et al., 2008). Highly competitive endurance athletes actually seem to have improved performance when using their attentional focus (e.g., focusing on technique or sensory systems) rather than allowing their minds to wander, suggesting cognitive demands may be high during intense exercise bouts (see Brick et al., 2014 for review). In fact, the use of associative cognitive strategies increases as pace or effort increases (Tammen, 1996), and high-level runners use specific strategies to control cognition during intense bouts (Brick et al., 2015). This differs from episodes of distraction or mind-wandering that are reported in less demanding exercise bouts (Brick et al., 2015) or in novice runners (Schücker et al., 2016).

Imaging studies (e.g., fNIRS and EEG) performed during locomotion support the hypothesis that movement can be cognitively demanding beyond simply automated motor control. Locomotion activates several brain regions, including prefrontal, parietal, and parahippocampal regions, which are often associated with executive functions, spatial navigation, and memory (Hamacher et al., 2015), and high-speed locomotion (e.g., running) increases the cognitive demands and associated neural activity (Suzuki et al., 2004; Hamacher et al., 2015). These studies suggest that locomotion in general, and high-speed locomotion specifically, engages several cognitive domains in ways that, over time, may alter brain structure, function, and connectivity.

To examine the relationship between competitive endurance exercise and the brain, we investigated brain function in expert endurance athletes and age-matched, non-athlete adults. We defined expertise in endurance sports following Baker et al. (2005) by identifying individuals based primarily on time spent in sport-specific training along with the achievement of high levels of performance (i.e., participation on a collegiate-level team indicating faster finishing times than age-matched non-experts). We compared groups using functional connectivity magnetic resonance imaging (fcMRI) assessed in the resting state, since functional brain networks have been previously suggested to play a role in the beneficial effects of exercise on cognition in older adults (see Burdette et al., 2010; Voss et al., 2010a,c). Resting-state fcMRI networks are observed using the blood oxygenation level-dependent (BOLD) signal when subjects are not actively performing a goal directed task, reflecting regionally distinct but functionally connected brain areas. Such network functional connectivity is thought to influence performance across many cognitive domains, and changes in fcMRI have been observed in the context of aging and in neurodegenerative diseases that are associated with diminished cognitive performance (Damoiseaux et al., 2008; Tomasi and Volkow, 2012). There is mounting evidence that exercise training in older adults can significantly increase functional connectivity within several fcMRI resting networks whose function is altered during both healthy and pathological aging (Burdette et al., 2010; Voss et al., 2010c). In addition, a single acute bout of exercise in young adults can lead to increased connectivity in sensorimotor fcMRI networks (Rajab et al., 2014).

Here, we investigated differences between expert endurance athletes and non-athlete young adult controls in three previously reported fcMRI resting-state networks that are either known to be affected by exercise, or are associated with cognitive domains influenced by physical activity (e.g., aspects of executive function and motor abilities): the default mode network (DMN), the frontoparietal network (FPN), and the motor network (MN). The DMN is the most widely studied fcMRI network (Ferreira and Busatto, 2013) and typically includes the medial prefrontal cortex, the hippocampus, the inferior parietal lobule, and the posterior cingulate cortex (Raichle et al., 2001; Buckner et al., 2008). The DMN is a network highly active at rest that is often associated with mind-wandering (Mason et al., 2007). Task-based deactivation of the DMN is associated with executive function, in addition to performance in other cognitive domains (Raichle et al., 2001; Greicius et al., 2003; Damoiseaux et al., 2008). Previous work has shown that, in older adults, exercise interventions affect DMN connectivity (Voss et al., 2010c). The FPN is a brain network that has been associated with executive functions, attention, and motor control (Seeley et al., 2007; Vincent et al., 2008; Pammi et al., 2012; Hsu et al., 2014), and often includes the anterior insula, the dorsolateral prefrontal cortex, the anterior prefrontal region, the dorsomedial superior frontal/anterior cingulate area, and the anterior inferior parietal lobule (Vincent et al., 2008). The MN is a functional brain network that has been associated with resting state connectivity involving the left and right motor cortices (Biswal et al., 1995). We hypothesized that individuals who engage in highly intense aerobic exercise (i.e., competitive endurance cross-country running) would differ in resting state functional connectivity in these networks compared with more sedentary, non-athlete controls due to the intense demands on executive functions that are intrinsically linked with such motor activities.

## Materials and Methods

### Sample

Twenty-two healthy young adults, 18–25 years of age, were recruited to participate in the study. All subjects were screened for any history of significant medical, neurological, or psychiatric injuries and disorders, which might affect brain structure or function. Additionally, participants were excluded if they had implants or dental work that would preclude magnetic resonance imaging (MRI) scanning. The participants included 11 male athletes and 11 age-matched male non-athlete controls. The athlete group was comprised of highly fit male distance runners, recruited from teams and clubs in the Tucson-metro areas currently competing in the NCAA junior college division. The age- and sex-matched non-athlete controls were recruited from the community and were individuals not currently participating in purposeful exercise and who have not participated in regular exercise or organized sports during the last year. The characteristics of the participants in the study are shown in **Table 1**. All participants provided informed written consent for the study, which was approved by the University of Arizona Institutional Review Board.

**Table 1:** Subject characteristics.

Variable	Controls	Athletes	*p*-Value
Sample size	11	11	–
Age, years (± SD)	20.64 (1.12)	21.55 (2.42)	0.278
Education (± SD)	14.00 (1.41)	13.55 (1.63)	0.494
Handedness (R/L)	8/3	10/1	0.586
MMSE (± SD)	29.36 (1.21)	29.00 (1.34)	0.511
BMI (± SD)	20.86 (3.34)	20.87 (2.28)	0.992
VO_2max_ ml O_2_ kg min^-1^ (± SD)^†^	39.43 (4.43)	62.14 (10.36)	5.44E-3
MET (± SD)	13.32 (0.71)	16.08 (0.76)	2.84E-8
PAQ (± SD)	3.96 (3.14)	25.69 (10.55)	3.01E-5


*All values are Mean (± Standard Deviation). ^†^Measured VO_*2*max_ collected from a subset of the original sample (*n* = 5 Athletes; *n* = 6 Controls). Key: R/L, Right/Left; MMSE, Mini-Mental State Exam; BMI, Body Mass index; VO_*2*max_, maximum oxygen consumption (ml O_*2*_ kg min^-*1*^); MET, Metabolic Equivalent (1 MET = 3.5 ml O_*2*_ kg^-*1*^ min^-*1*^); PAQ, Total score of Self-Reported Physical Activity Questionnaire (Sum of household, leisure and sport scores).*

### Aerobic Fitness Assessments

Three measures were used to assess levels of exercise engagement and overall aerobic fitness. We first grouped participants based on their reported participation in organized endurance sports (cross-country runners were placed in the athlete group; controls were placed in the non-athlete control group). Second, we used a physical activity questionnaire (PAQ) to determine total self-reported amounts of physical activity on a continuous scale that incorporates information on the types and intensity of activities, as well as hours over time periods spent (Voorrips et al., 1991). Third, we calculated an estimated fitness score (in metabolic equivalents or METs) based on anthropometrics and exercise participation following Jurca et al. (2005). Briefly, this score, which differs from questions in the PAQ, combines a self-reported ordinal scale of exercise engagement (i.e., subjects placed in one of five levels of time spent in aerobic activity each week) with body mass index (BMI), age, sex, and resting heart rate (collected after 5 min of quiet rest) to estimate maximal aerobic capacity or VO_2max_, expressed in METs (where 1 MET = 3.5 ml O_2_ kg^-1^min^-1^). While the PAQ and MET values are correlated in our sample (*r*^2^ = 0.58; *p* < 0.001), 42% of the variance in PAQ is not explained by estimated VO_2max_, suggesting engagement and cardiovascular fitness are measuring different aspects of exercise. Finally, a subset of our sample (*n* = 5 athletes; *n* = 6 non-athletes) completed a graded exercise test to quantitatively measure VO_2max_. We used the Costill/Fox protocol (Costill and Fox, 1969) to determine maximum aerobic capacity. This protocol consists of changes in both speed and incline, and is considered appropriate for determining VO_2max_ in both untrained and in highly trained aerobic athletes (Kang et al., 2001).

### Imaging Methods

#### MRI Procedures

Magnetic resonance imaging scans were acquired on a long-bore 3.0T GE Signa scanner (General Electric, Milwaukee, WI, USA). Resting state fcMRI scans were obtained while participants were resting comfortably in the scanner with eyes open for a period of 6 min consisting of 150 volumes using a gradient-echo echo-planar imaging sequence (TE = 30 ms, TR = 2400 ms, FA = 90°, FOV = 25.6 cm × 25.6 cm, matrix = 64 × 64, 32 contiguous 3.8 mm slices). The first four image volumes were discarded to allow the BOLD signal to reach steady state. T1-weighted 3D SPGR anatomical MRI scans were acquired during the same scanning session (TE = 2.0 ms, TR = 5.3 ms, FA = 15.0°, FOV = 25.6 cm × 25.6 cm, matrix = 256 × 256, slice thickness = 1.0 mm).

#### Image Analysis

Images were processed using the CONN functional connectivity toolbox v15.a (Whitfield-Gabrieli and Nieto-Castanon, 2012^1^) and SPM12^2^. Preprocessing procedures involved inter-frame motion correction and unwarping, slice timing correction, co-registration of the functional images to their respective anatomical MRI images, structural segmentation and spatial normalization, and functional spatial normalization. A motion and outlier scrubbing step was included using the Artifact Detection Tools software (ART^3^), to identify scans affected by movement-related artifacts, following which the functional images were smoothed using a Gaussian kernel of 8 mm full-width at half maximum.

Several potentially confounding sources of signal variation were removed by linear regression. These included six head motion parameters, signals from the white matter (WM) and cerebrospinal fluid, and the outlier scans detected during the scrubbing process. In the CONN toolbox, motion outliers are identified using ART, where the combination of translational and rotational voxel displacements are derived from the frame-to-frame trajectory of six control points placed at the center of the six faces of the brain image bounding box. For signal outliers, the mean signal across the entire time series was computed and Z-transformed. Frames were classified as outliers if either the composite motion from the combination of translational and rotational displacements exceeded 2mm or the global mean signal exceeded 9 SD or both (Whitfield-Gabrieli and Nieto-Castanon, 2012; Ely et al., 2016). With this approach, the number of outlier volumes detected ranged from 0 to 4.6% of the total fcMRI volumes per participant. In addition, the groups did not differ in the composite average, maximum, and absolute maximum measures of head motion [*t*(20) ≤ 0.53, *p*’s ≥ 0.60]. Importantly, the CONN software uses an anatomical component-based noise correction method (aCompCor) (Behzadi et al., 2007) that does not rely on average global signal regression to remove sources of noise in the BOLD time series data (Whitfield-Gabrieli and Nieto-Castanon, 2012), and instead derives the principal components from the WM and cerebrospinal fluid regions using the high resolution T1 scan. As such, observed anti-correlations in the fcMRI connectivity analyses are not attributable to potential biases from global signal regression artifacts (Chai et al., 2012, 2014). Finally, a temporal band pass filter (0.008 to 0.09 Hz) was applied to this residual BOLD signal.

**FIGURE 1:**
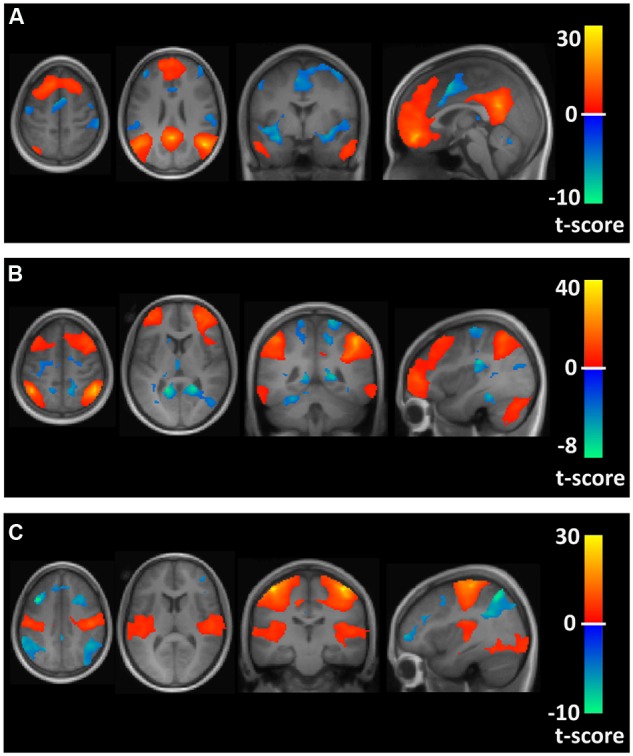
**Axial, coronal, and sagittal views of spatial maps for each resting state network (orange) and the corresponding anti-correlations (blue) for the conjunction of the seed region maps averaged across all 22 participants.** **(A–C)** Show the default mode network (DMN), frontoparietal network (FPN), and motor network (MN), respectively.

The time course of this filtered BOLD signal from 4 mm radius spherical regions for regional seeds in the FPN, DMN, and MN, defined by Montreal Neurological Institute (MNI) brain coordinates as reported by Vincent et al. (2008), were correlated with every other brain voxel’s time course to obtain brain maps of correlation coefficients for each participant. For the FPN, seed regions in the left and right anterior prefrontal cortex (MNI coordinates: -36, 57, 3 and 36, 57, 3) and inferior parietal lobule (MNI coordinates: -44, -52, 54 and 48, -50, 52) were used. For the DMN, seed regions were localized in the posterior cingulate (MNI coordinates: 0, -53, 26), medial prefrontal cortex (MNI coordinates: 0, 54, -4) and left and right inferior parietal lobule (MNI coordinates: -46, -48, 36 and 50, -62, 32). Seed regions for the MN included seeds selected from the left and right motor cortex (MNI coordinates: -42, -25, 63 and 42, -25, 63). Brain maps showing the average correlation and anti-correlation networks for the conjunction of the seed region maps across all 22 participants for the FPN, DMN, and MN are shown in **Figure 1**.

### Statistical Analysis

The participant maps of the correlations between the fcMRI seed regions and all voxels of the brain were entered into a second-level group analysis, using a two sample *t*-test to test for between group differences where either athletes showed significantly greater connectivity values than controls, or where controls showed significantly greater values than athletes. Group differences were tested for connectivity values (fisher transformed correlation coefficients). The average functional connectivity values for each participant from suprathreshold voxel clusters were extracted to test within-group connectivity differences from zero with one-sample *t*-test and their relation to measures of physical and estimated metabolic activity with linear regressions. For all fcMRI connectivity values, significance was taken by applying both an uncorrected *p* < 0.001 for magnitude and an FDR corrected *p* < 0.05 for spatial extent. Group differences in subject characteristics were tested with a *t*-test and the Fisher’s Exact test, where appropriate. Unthresholded statistical maps have been uploaded to NeuroVault.org and can be viewed at http://neurovault.org/collections/1508/.

## Results

The two groups (athletes and non-athlete controls) did not differ in age, education, or BMI (see **Table 1**). As expected, the groups did differ significantly in measures of aerobic fitness and physical activity. Although available for only a subset of the participants, the athlete group had significantly higher measured VO_2max_ values compared to the non-athlete controls (**Table 1**). Furthermore, we used these measured values to evaluate the estimated VO_2max_ values (METs) for subsequent analyses with the brain imaging data. We found the measured VO_2max_ values and the estimated values were strongly correlated (*r* = 0.73; *p* = 0.01), and the athletes had significantly higher estimated VO_2max_ values compared to the controls. Finally, self reported levels of physical activity were significantly higher in the athlete group compared to controls (**Table 1**).

**FIGURE 2:**
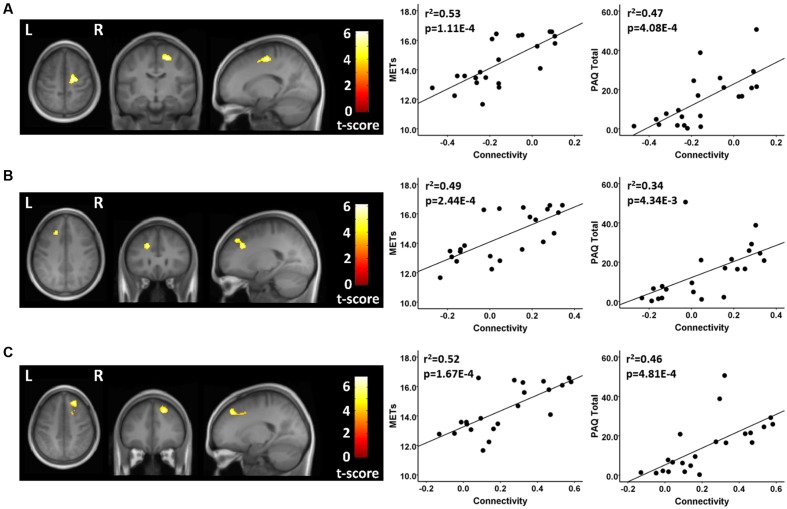
**Resting state fcMRI differences between athletes and controls in the FPN.** **(A – left panel)** Cluster in the vicinity of the right superior frontal/supplementary motor region (MNI: 18, -14, 56) where endurance athletes had greater functional connectivity with the left anterior seed of the FPN. **(A – right panel)** Scatterplots showing the relation between connectivity values at this cluster (MNI: 18, -14, 56), and the MET and PAQ measures. **(B – left panel)** Cluster in the vicinity of the left superior/mid frontal region (MNI: -20, 28, 34) where endurance athletes had greater functional connectivity with the left anterior seed of the FPN. **(B – right panel)** Scatterplots showing the relation between connectivity values at this cluster (MNI: -20, 28, 34), and the MET and PAQ measures. **(C – left panel)** Cluster in the vicinity of the right superior/mid frontal region (MNI: 22, 36, 46) where endurance athletes had greater functional connectivity with the right parietal seed of the FPN. **(C – right panel)** Scatterplots showing the relation between connectivity values at this cluster (MNI: -20, 28, 34), and the MET and PAQ measures. MNI, Montreal Neurological Institute coordinates in the x, y, and z planes for cluster peak value; MET, Metabolic Equivalent; PAQ, Total score of Self-Reported Physical Activity Questionnaire.

There were several between-group differences in functional connectivity involving each of the three resting state fcMRI networks. In testing where the athletes were greater than the controls using seeds in the FPN, the two groups had significantly different patterns of connectivity between two network seeds and three frontal brain regions. The athletes had significantly higher functional connectivity values than controls between a right parietal seed in the FPN and a region in the vicinity of the right superior/mid frontal region (**Figure 2**; **Table 2**). This difference reflected a significant positive connectivity value during the resting state in the athlete group and no significant value from zero in the non-athlete control group. The athletes also had significantly greater connectivity than controls between a seed in the left anterior FPN and an area in the vicinity of the left superior/mid frontal regions (**Figure 2**; **Table 2**). This between-group difference was reflected by a strong positive connectivity value between regions in the athletes and a significant anti-correlation between regions in the non-athlete control group. Finally, the athletes differed significantly in connectivity values from controls between a seed region in the left anterior FPN and an area in the vicinity of the right superior frontal and supplementary motor regions. The between-group difference reflected an anti-correlation between regions in the non-athlete control group, and no significant values from zero between these regions in the athlete group (**Table 2**). For the FPN, no significant regional differences were observed where the controls were greater than the athletes.

**Table 2:** Frontoparietal network (FPN) connectivity analysis.

Seed region	Contrast	Region of difference	Cluster size (Voxels)	Athletes	Controls	Peak *p*-uncorrected	Cluster p-FDR
FPN-left anterior seed (-36, 57, 3)	Athletes > Controls	Right superior front/supplementary motor (18, -14, 56)	183	-0.016 ± 0.11	-0.28 ± 0.09^∗∗∗^	0.000003	0.020016
	Athletes > Controls	Left superior/mid frontal (-20, 28, 34)	133	0.21 ± 0.12^∗∗∗^	-0.085 ± 0.12^∗^	0.000009	0.034096
FPN-right parietal seed (48, -50, 52)	Athletes > Controls	Right superior/mid frontal (22, 36, 46)	220	0.40 ± 0.15^∗∗∗^	0.05 ± 0.09	0.000001	0.012157


*Mean ± SD connectivity values for each group differing from zero at ^∗^*p* < 0.05; ^∗∗∗^*p* < 0.001; numbers in parentheses reflect Montreal Neurological Institute coordinates in the x, y, and z planes reflecting the peak voxel value for defining the seed region and for the region of difference cluster.*

**FIGURE 3:**
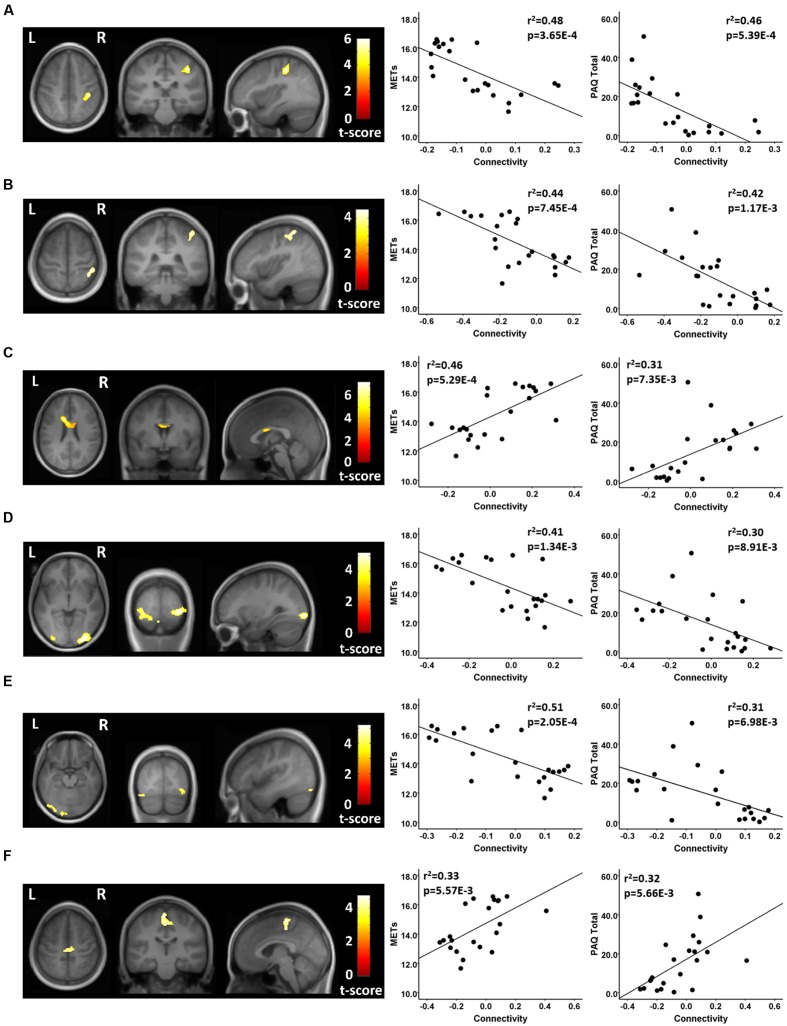
**Resting state fcMRI differences between athletes and controls in the DMN.** **(A – left panel)** Cluster in the vicinity of the right post-central region (MNI: 38, -30, 48) where endurance athletes had lower functional connectivity with the medial prefrontal cortex seed of the DMN. **(A – right panel)** Scatterplots showing the relation between connectivity values at this cluster (MNI: 38, -30, 48), and the MET and PAQ measures. **(B – left panel)** Cluster in the vicinity of the right post-central/superior parietal region (MNI: 38, -48, 62) where endurance athletes had lower functional connectivity with the posterior cingulate seed of the DMN. **(B – right panel)** Scatterplots showing the relation between connectivity values at this cluster (MNI: 38, -48, 62), and the MET and PAQ measures. **(C – left panel)** Cluster in the left frontal white matter (MNI: -14, 14, 26) where endurance athletes had greater functional connectivity with the medial prefrontal cortex seed of the DMN. **(C – right panel)** Scatterplots showing the relation between connectivity values at this cluster (MNI: -14, 14, 26), and the MET and PAQ measures. **D – left panel)** Cluster in the region of the right occipital cortex (MNI: 30, -92, -4) where endurance athletes had lower functional connectivity with the left parietal seed of the DMN. **(D – right panel)** Scatterplots showing the relation between connectivity values at this cluster (MNI: 30, -92, -4), and the MET and PAQ measures. **(E – left panel)** Cluster in the vicinity of the left occipital cortex (MNI: -2, -94, -20) where endurance athletes had lower functional connectivity with the left parietal seed of the DMN. **(E – right panel)** Scatterplots showing the relation between connectivity values at this cluster (MNI: -2, -94, -20), and the MET and PAQ measures. **(F – left panel)** Cluster in the vicinity of the left paracentral region (MNI: -2, -24, 52) where endurance athletes had greater functional connectivity with the left parietal seed of the DMN. **(F – right panel)** Scatterplots showing the relation between connectivity values at this cluster (MNI: -2, -24, 52), and the MET and PAQ measures. MNI, Montreal Neurological Institute coordinates in the x, y, and z planes for cluster peak value; MET, Metabolic Equivalent; PAQ, Total score of Self-Reported Physical Activity Questionnaire.

Using seeds in the DMN, the athletes and non-athletes differed in connectivity between network seed regions and six other brain regions. Using a medial prefrontal seed in the DMN, the athletes showed significantly different functional connectivity with two clusters of voxels. First, testing where the controls were greater than the athletes, the athletes had a significant anti-correlation between the medial prefrontal seed region and a region in the vicinity of the right post-central gyrus, while the non-athlete controls did not show a significant value from zero in this region (**Figure 3**; **Table 3**). Testing where the athletes were greater than the controls, the medial prefrontal seed showed a positive connectivity value with a subcortical region in the frontal WM of the athletes, while the control group had a significant anti-correlation between the medial prefrontal seed and this WM region (**Figure 3**; **Table 3**).

**Table 3:** Default mode network (DMN).

Seed region	Contrast	Region of difference	Cluster size (Voxels)	Athletes	Controls	Peak *p*-uncorrected	Cluster *p*-FDR
DMN-medial prefrontal seed (0, 52, -6)	Athletes > Controls	Frontal WM -14, 14, 26)	371	0.16 ± 0.11^∗∗∗^	-0.11 ± 0.09^∗∗^	<0.000001	0.000394
	Controls > Athletes	Right post-central (38, -30, 48)	269	-0.15 ± 0.05^∗∗∗^	0.06 ± 0.11	0.000004	0.001203
DMN-posterior cingulate seed (0, -53, 26)	Controls > Athletes	Right precentral/sup parietal (38, -48, 62)	189	-0.26 ± 0.13^∗∗∗^	0.02 ± 0.13	0.000119	0.045085
DMN-left parietal seed (-48, -62, 36)	Athletes > Controls	Left paracentral (-2, -24, 52)	312	0.07 ± 0.14	-0.17 ± 0.11^∗∗∗^	0.000064	0.002123
	Controls > Athletes	Right lat occipital (30, -92, -4)	462	-0.15 ± 0.16^∗∗^	0.11 ± 0.09^∗∗^	0.000022	0.000124
	Controls > Athletes	Left lat occipital (-2, -94, -20)	436	-0.16 ± 0.12^∗∗∗^	0.09 ± 0.09^∗∗^	0.00038	0.000124


*Mean ± SD connectivity values for each group differing from zero at ^∗∗^*p* < 0.01; ^∗∗∗^*p* < 0.001; numbers in parentheses reflect Montreal Neurological Institute coordinates in the x, y, and z planes reflecting the peak voxel value for defining the seed region and for the region of difference cluster.*

In testing where the controls were greater than the athletes, using a seed in the posterior cingulate region of the DMN, the athletes showed a significant anti-correlation with an area in the vicinity of the right post-central and superior parietal region, while the non-athlete controls did not differ from zero in the association with this region (**Figure 3**; **Table 3**). Testing where the controls were greater than the athletes, with a seed in the left parietal region of the DMN, the athletes had significant anti-correlations with regions in the vicinity of the right and left lateral occipital cortex, while the non-athlete adults had significant positive connectivity values with these two regions (**Figure 3**; **Table 3**). Using this same left parietal seed region of the DMN, but testing where the athletes were greater than controls, the non-athlete adults had a significant anti-correlation with a region in the vicinity in the left paracentral area, while the athletes did not show a significant value from zero in association with this region (**Figure 3**; **Table 3**). There were no other significant regional differences between groups for the relation between the DMN seeds and other brain regions.

**FIGURE 4:**
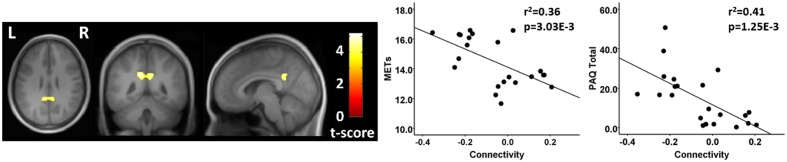
**Resting state fcMRI differences between athletes and controls in the MN.** **(left panel)** Cluster in the vicinity of the posterior cingulate (MNI: -12, -46, 32) where control participants had greater functional connectivity with the right motor cortex. **(right panel)** Scatterplots showing the relation between connectivity values at this cluster (MNI: -12, -46, 32), and the MET and PAQ measures. MNI, Montreal Neurological Institute coordinates in the x, y, and z planes for cluster peak value; MET, Metabolic Equivalent; PAQ, Total score of Self-Reported Physical Activity Questionnaire.

Finally, testing where the controls were greater than athletes using seeds in the MN, there was a significant between group difference in connectivity between a seed in the right MN and a region in the vicinity of the posterior cingulate cortex. This between group difference was due to a significant anti-correlation in the athlete group and no significant value from zero in the non-athlete control group (**Figure 4**; **Table 4**). There were no significant regional differences where the athletes were greater than controls in association between the MN seeds and any brain region.

**Table 4:** Motor network (MN) connectivity analysis.

Seed region	Contrast	Region of difference	Cluster size (Voxels)	Athletes	Controls	Peak *p*-uncorrected	Cluster *p*-FDR
Right motor cortex (42, -25, 63)	Controls > Athletes	Posterior cingulate (-12, -46, 32)	292	-0.18 ± 0.10^∗^^∗^^∗^	0.06 ± 0.1	0.000021	0.001678


*Mean ± SD connectivity values for each group differing from zero at ^∗∗∗^*p* < 0.001; numbers in parentheses reflect Montreal Neurological Institute coordinates in the x, y, and z planes reflecting the peak voxel value for defining the seed region and for the region of difference cluster.*

For each of these functional connectivity differences between athletes and non-athlete young adult controls, there were significant linear associations between the connectivity values extracted from each region that was related to the corresponding network seed and self-reported physical activity scores (**Figures 2–4**). Similarly, significant linear associations were found between regional values at each of these regions and the composite MET scores (**Figures 2–4**). Slopes for the best-fit linear regression lines were negative when between-group differences were reflected by stronger regional anti-correlations in the athlete group, and were positive when the between-group regional differences were driven by stronger positive regional connectivity values in the athlete group.

## Discussion

Our results suggest that engagement in high levels of aerobic activity in young adulthood is associated with differences in resting state functional connectivity in networks known to be linked to executive function and motor control compared with more sedentary individuals. This is the first study, to our knowledge, to show that high levels of participation and expertise in endurance running are reflected by differences in the resting state fcMRI networks of young adults. In addition, there appears to be a dose-response effect of participating in highly intense activity on resting-state functional connectivity. The linear regressions showed that time spent in highly aerobic activities, and higher levels of aerobic fitness (estimated VO_2max_), were associated with greater connectivity values across the sample. These findings are consistent with those observed in studies of older adults that suggest aerobic exercise and fitness are associated with neuroplasticity in resting state networks (Burdette et al., 2010; Voss et al., 2010a,c).

Our results show that differences between expert endurance athletes and non-athlete young adults included both enhanced positive connectivity, as well as anti-correlations between regions. The detection of between-group differences suggests that athletes may stress cognitive domains in ways that reflect underlying connections across key brain regions. Strengthened connectivity may be associated with histories of co-activation of linked regions (Guerra-Carrillo et al., 2014), suggesting high-level endurance running may engage these connected regions, while enhancing connectivity through increased aerobic activity. Previous researchers have hypothesized that negative associations between resting state regions may reflect the resolution of conflicting cognitive demands, and anti-correlations between the DMN and task-positive regions have been linked to improved cognitive performance in recent work (see Fox et al., 2006; Keller et al., 2015). Thus, anti-correlations may reflect out of phase functional associations between resting state network brain regions or with those extra-network brain areas engaged during overt cognitive activity.

While there is some debate over the cognitive implications of between group differences in resting state functional connectivity networks (Cole et al., 2010), the increased connectivity between select brain regions and the FPN, the DMN, and the MN is consistent with links between motor control and the implicit cognitive demands of high intensity endurance running activity. As described earlier, movement, especially at high speeds, taxes not only motor control, but can also engage executive functions, spatial navigation, and memory abilities. Over time, these linked cognitive demands may have beneficial effects on brain structure and function. In fact, recent studies have shown that highly fit and competitive young adult athletes perform better on tests of executive function and processing speed in non-sport specific cognitive tasks, suggesting exercise-based improvements may be generalizable to cognitive demands during daily life (Voss et al., 2010b; Chaddock et al., 2011). For example, Chaddock et al. (2011) showed that athletes perform better than non-athletes on a virtual street-crossing task that requires multi-tasking while walking. Perhaps the kinds of changes that occur in resting state fcMRI of athletes demonstrated in our study supports this type of high performance executive function *during* locomotion. As such, it would be expected that the brain regions sub-serving these cognitive functions may show enhanced patterns of functional connectivity in response to frequent and sustained endurance running activity.

### Connectivity Patterns

We found two overarching patterns that distinguished the expert endurance athletes from non-athletes. First, we found support for enhanced connectivity between the FPN and regions that have been associated with frontal mediated cognitive functions, including aspects of working memory in the athlete group (see Klingberg et al., 1997; du Boisgueheneuc et al., 2006). Since the FPN is generally associated with executive function, including planning of motor control (Vincent et al., 2008; Pammi et al., 2012), it is possible that between-group connectivity differences reflect, in part, executive function demands during endurance running. Interestingly, improvements in this cognitive domain are often associated with increased physical activity in older adults, and the findings in older adults combined with our results provide initial support for the hypothesis that aerobic exercise enhances connectivity in brain regions linked to executive functions across the lifespan (see Voss et al., 2010c).

Second, we found strong patterns of anti-correlations between the DMN of athletes and brain regions often associated with functions that may be especially stressed during endurance running. These functions include motor control, somatosensory needs which may be associated with foot strike during running, and visual processing (Nolte, 2009), which may be needed for planning foot placement and for navigation. In addition, in athletes, a seed region in the MN was strongly anti-correlated with the posterior cingulate, a key area of the DMN. The DMN is a network highly engaged at rest, and enhanced cognitive performance is associated with both DMN deactivation during tasks, and with resting-state anti-correlations between the DMN and task-positive networks (Gusnard and Raichle, 2001; Greicius and Menon, 2004; Keller et al., 2015). The pattern of anti-correlations found in the athlete group is consistent with the hypothesis that DMN deactivation may enhance performance in cognitive domains required of high-level endurance running.

In addition to these two key patterns, we found a significant positive connectivity value between a seed region in the DMN and a nearby subcortical region that included mainly WM. Previous work has suggested that fMRI signals in WM should not occur due to both relatively lower blood flow compared with gray matter, and the possibility that fMRI signals occur in response to post-synaptic signals in gray matter (see Logothetis et al., 2001; Logothetis and Wandell, 2004). However, there is mounting evidence that the vasculature in WM can lead to a measureable BOLD fMRI signal, mainly during task-based activities (Maldjian et al., 1999; Tettamanti et al., 2002; Weber et al., 2005; Mazerolle et al., 2010; Gawryluk et al., 2014). Our results may be related to greater WM vasculature density and blood flow in selective frontal brain regions in endurance athletes compared with non-athletes, allowing us to detect a relatively stronger WM signal at rest. Supporting this hypothesis, recent work has shown that exercise can increase vascular density in the brain (Bullitt et al., 2009). Further study of highly athletic individuals is needed to address the question of differences in WM signal with fcMRI and to gain a better understanding of potential underlying mechanisms.

Finally, we found two differences that suggest greater anti-correlations in our non-athlete young adults than athletes. Specifically, the non-athlete group had greater anti-correlations between the left anterior seed in the FPN and an area near the right superior frontal and supplementary motor regions, areas often associated with motor control, and significant anti-correlations between the DMN and a region in the vicinity of the left paracentral region, while athletes did not show significant connectivity values in either case. These findings may reflect regional differences in the reliance on the FPN and DMN for overt cognitive activity in the non-athletes compared to athletes. Further research is needed to better understand the source of such regional differences and how they are related to differences in cognitive performance.

Overall, functionally connected regions found in this study are consistent with the needs of athletes during high intensity running. For example, high-speed running entails the combination of complex, sequential motor tasks, multi-tasking, working memory, and object recognition. We also found evidence that patterns may be driven by time spent in intense aerobic activity. In all of the patterns described above, connectivity strength (both positive and negative) was highly correlated with measures of aerobic fitness and self-reported physical activity. Furthermore, these connectivity findings were not influenced by the four left-handed participants in our cohort, as there was no difference between the groups in handedness and all relations to MET and physical activity scores remained significant when the left-handers were removed from the regression analyses (4.86E-5 ≤ p ≤ 0.038). Although experimental interventions would be needed to infer causation, these results do suggest that between-group differences in fcMRI connectivity are related to inter-individual differences in engagement in endurance exercise. Future work should focus on longitudinal assessments of endurance exercise training and resting state connectivity to determine the amount and type of activity that most strongly impacts changes in functional connectivity and their relation to changes in cognitive and motor skill.

### Possible Mechanisms for Between-Group Connectivity Differences

Recent work suggests that connectivity strength reflects a history of co-activation of functionally connected regions (see Lewis et al., 2009; Raichle, 2011; Buckner et al., 2013; Keller and Just, 2016), and patterns of resting state connectivity may indicate a history of task-dependent activity (see Duan et al., 2012). We suggest that between-group differences in our study are likely due to functionally covarying regions during endurance running that, over time, lead to strengthened functional connections within resting state networks and specific functional associations with task related extra-network regions. Our observations showing the association of correlation strength between brain regions and time spent in physical activity provides some support for this interpretation.

Over the short term, several studies have shown that learning induces acute changes in resting state fcMRI (Keller and Just, 2016). For example, after a short (11 min) motor training session, subjects showed strengthened connections in the FPN compared with a control group (Albert et al., 2009). Woolley et al. (2015) recently showed that one session of training on a virtual water maze led to an increase in resting state functional connectivity between the hippocampus and the dorsal caudate, and the magnitude of the change in connectivity was correlated with future task performance. Finally, recent work suggests that training-induced changes in resting state fcMRI activity are related to underlying structural effects that may provide a window into how and why these networks impact cognitive performance (Keller and Just, 2016). Using a virtual spatial navigation training task, Keller and Just (2016) found that one training session led to increased resting state connectivity between the hippocampus and cortical areas that was associated with changes in diffusivity in the hippocampus.

Longer-term training that stresses specific cognitive domains may result in lasting and stable changes in resting state fcMRI, a result that has been demonstrated by comparing individuals considered experts in specific behaviors with healthy controls. For example, Jang et al. (2011) examined differences in the DMN of long-term practitioners of meditation compared to healthy controls. They found increased functional connectivity in the anterior medial prefrontal cortex of meditation subjects, an area that is related to self-relevant mental simulation (Jang et al., 2011). In chess grand masters, high levels of intrinsic functional connectivity in the DMN were associated with greater levels of DMN deactivation during cognitive tasks (Duan et al., 2012). Expert musicians show several distinct differences in resting state connectivity patterns compared with non-musicians (Fauvel et al., 2014). These differences include enhanced connectivity between clusters in the cingulate gyrus, the right prefrontal cortex, and the left temporal pole (Fauvel et al., 2014). In professional badminton players, a sport that requires complex motor control, functional connectivity between frontal regions and the left superior parietal region was altered compared with healthy non-athlete controls (Di et al., 2012). Finally, a recent study of world-class gymnasts also found effects of long-term motor training on resting state functional connectivity (Wang et al., 2016). Gymnasts had reduced connectivity within what the authors term functional modules compared with controls (the cerebellum, fronto-parietal, and cingulo-opercular regions), which they attributed to enhanced neural efficiency in these areas (Wang et al., 2016).

Taken together these studies suggest that, over time, co-activation of brain areas can enhance functional networks at rest and that the strength of resting state connectivity can reflect long-term training in a variety of activities. Within the context of our results, long-term training for endurance running may result in co-activation of specific brain regions within and related to networks associated with motor control and executive function. Over time, expert athletes may develop enhanced connectivity that may in turn lead to greater efficiency in performing the implicit motor and cognitive tasks associated with long-distance endurance running. Further research is needed to address this question directly with sensitive measures of cognitive and motor skills in both young and older adult athletes compared to non-athlete controls.

## Conclusion

Our results show, for the first time, clear differences in resting state functional connectivity between expert endurance athletes and healthy age-matched non-athletes. These differences may arise in response to the cognitive demands of long distance running combined with aerobic exercise. It is possible that differences in resting state fcMRI activity may improve aerobic athletic performance by allowing more efficient execution of cognitive and motor demands during highly intense activity. Given the correlation between cardiovascular fitness and exercise participation variables and connectivity, it is possible that an examination of elite athletes would reveal even greater connectivity differences. This is an area that should be explored in future work. In addition, the cognitive demands of high intensity endurance exercise may also lead to differences in performance on activities that are unrelated to sports. Previous work has in fact identified greater performance on non-sport specific cognitive tasks in competitive athletes (Voss et al., 2010b; Chaddock et al., 2011). Future work should investigate resting state functional connectivity as a predictor of both athletic performance in young adults and cognitive performance on non-sport specific cognitive tasks.

While we focused here on expert endurance athletes, most studies of the effects of aerobic exercise on brain structure and function include recreational athletes in their sample. Thus, a limitation of our study is that we do not have a recreational athlete group that would allow us to more clearly distinguish the neural effects of expertise compared with general effects of lower-level exercise participation. While we believe there is great value in examining the extremes of athletic engagement (i.e., expert vs. sedentary), there is a need for future work to examine resting state functional connectivity in recreational young adult runners. These studies, combined with our data, will help us fully understand the nature of the dose-response relationship between aerobic activity and resting state connectivity and may help us determine the level of athletic engagement needed to alter functional networks. In addition, these types of studies will help us identify whether sedentary behavior itself is a driver of some of these changes in functional networks. It will also be important for future studies to include larger samples with both men and women, as well as expanded assessments of physical fitness, lifestyle histories, and cognitive functions across the age-range from young to older adults.

Finally, we believe that exercise-induced neuroplasticity in young adults should be investigated in relation to brain aging and the potential to reduce vulnerability to cognitive aging and the risk for neurodegenerative disease. There is growing evidence from retrospective studies that exercise at younger ages can have protective effects on brain aging (Rovio et al., 2005; Kulmala et al., 2014; Tolppanen et al., 2014). In older adults, self-reported early adult and mid-life exercise is correlated with enhanced global cognition (Gill et al., 2015), memory (Richards et al., 2003), and executive function (Sabia et al., 2009). Additionally, mid-life physical activity has been associated with reduced risk of developing neurodegenerative diseases (Rovio et al., 2005). Enhanced connectivity may occur in response to cognitive demands during exercise, yet the strengthened connections may improve executive function more broadly, allowing for improved cognitive function later in life. Lifelong physical activity may be an important element of successful aging and strengthened resting state connectivity could reflect a mechanism for the protective effects of physical activity. Together, our findings suggest that more detailed investigations of exercise-induced neuroplasticity in young adults may help us better understand how lifelong healthy behaviors can improve quality of life in older adults.

## Author Contributions

DR and GA conceived of and designed the study. MF, KH, G-AT, DR, and GA collected data. PB, TT, DR, and GA analyzed data. All authors contributed to the writing and editing of the final manuscript.

## Conflict of Interest Statement

The authors declare that the research was conducted in the absence of any commercial or financial relationships that could be construed as a potential conflict of interest.

## Footnotes

1 http://www.nitrc.org/projects/conn

2 http://www.fil.ion.ucl.ac.uk/spm/

3 https://www.nitrc.org/projects/artifact_detect/
